# Abstracts from Foreign Journals

**Published:** 1902-07

**Authors:** S. J. J. Harger

**Affiliations:** Philadelphia, Pa.


### ABSTRACTS FROM FOREIGN JOURNALS.

### FRENCH.

Under the Direction of S. J. J. Harger, V.M.D.,
PHILADELPHIA, PA.

Local Anæsthesia. Under certain conditions, as in certain operations, as well as in track horses, a prolonged local analgesia of a nerve trunk may be desirable. Morphine seems to have a local effect similiar to that of cocaine, but its action is more prolonged. For this purpose Pécus recommends the following solution for one injection:

| | |
|---|---|
| Hydrochlorate of Cocaine . . . . . | gr. 15 |
| Hydrochlorate of Morphine . . . . . | gr. 10 |
| Distilled Water . . . . . . . . | 5 grammes. |

—*Rec. de Med. Vet.*

Syndactylism of the Median Digits in Artiodactyla. Lesbre, from his studies, concludes as follows:

1. Syndactylism, or fusion of the middle digits, may occur in the ox, pig and all other artiodactyla in which these digits are separated.

2. Although in ruminants this condition may be considered as an extension of the process of fusion of the metacarpal and metatarsal bones of the canon, nevertheless it is much less frequent in this species than in the pig.

3. This fusion may be limited to the region of the first phalanx; sometimes it may be complete, producing apparently a single digit.

4. This syndactylism in the pig is hereditary in a high degree, and has permitted Vasilescu, of Roumania, to create a soliped family of pigs. If this result has not been observed in the larger ruminants it may be due to the fact of insufficient observations and because animals so affected are slaughtered when young.—*Annales de Med. Vet.*

Dermosapol. This preparation is a mixture of oil or other fatty body, lanolin, and paraffine, to which is added a sufficient quantity of an alkali to produce complete saponification; besides, other preparations which it is desired to be absorbed by the skin may be incorporated. This mixture, which is useful in dermatology, gynecology and surgery, may be combined with lysol, phenic acid, corrosive sublimate, etc., to disinfect the hands and the skin of the field of operation.—*Rev. Pharmac.*

Honthin. This is a combination of tannin and albumin analogous to tanalbin. It is dissolved in the stomach, but is soluble in the small intestine on account of the alkaline medium.

Honthin is a greenish-brown powder, tasteless, odorless, insoluble in water, but soluble in alcohol and in alkaline solutions. It is employed in diarrhœa in infants and in enteritis.

The dose for an infant is 25 to 50 centigrammes, repeated four or five times daily; for an adult, 1 to 2 grammes.

This preparation is non-poisonous, and does not produce malaise, vomiting, or gastric pain.—*Wien. Klin. Wochens.*

Experiments upon the Excitability of the Spinal Cord. The gray substance of the cerebrum is excitable and reacts to mechanical and electrical excitants. Vitzon experimented upon the spinal cord of birds and horses to determine whether the gray substance is excitable, which was generally believed not to respond to stimulations.

In birds the lumbar portion of the cord was exposed, and when the hemorrhage was arrested, about an hour afterward, mechanical excitation produced muscular movements of the posterior parts, especially the tail.

Similar experiments were made upon a horse previously anæsthetized with chloral. The lumbar portion of the cord was exposed and cut transversely. Mechanical irritation produced no effect, due to the loss of blood during the operation, but electrical stimulation of the cephalic, as well as the caudal stumps of the cord, produced tetanic contractions.

The author concludes from his experiments that the gray substance of the spinal cord, like that of the cerebral cortex, is excitable by mechanical and electrical irritation.—*Acad. des Sc. de Paris.*

---

### GERMAN.

Clinical Notes on the Treatment of Various Diseases in Dogs. By Professor Albrecht, Munich. In a previous communication I referred to some observations on the use of protargol in veterinary practice. Recently I have again tried the remedy in a few local conditions in dogs, and give here the results obtained in these cases. The new indications for which I have utilized protargol comprises otitis externa and preputial catarrhs in dogs. In four cases the otitis was acute and in one of chronic character. In the former the disease had been present only for six or eight days before it came under observation. In the latter it had existed for four weeks. All the patients manifested a very severe itching and had a purulent offensive discharge from the external ear. For the treätment I made use of a 3 per cent. solution of protargol, of which one to two c. cm. were instilled into the external auditory meatus after previous careful cleansing. The meatus was then closed by pressure of the fingers upon the ear for three minutes, and not by plugging with gauze. Before the application of the protargol solution the ear was cleansed by means of a thoroughly sterilized piece of sponge in the grasp of a forceps, the sponge being thoroughly saturated with a 1 per cent. lysol solution. In all instances a cure occurred after ten days' treatment.

A fox terrier had suffered for some time with preputial catarrh. After the animal had been treated for sixteen days with injections of a 1 per cent. solution of zinc sulphate without improvement I directed the daily injection of a 3 per cent. protargol solution. Of this 5 c. cm. were employed each time, the preputial cavity being held closed for three to five minutes, and the fluid disseminated over the entire interior by means of gentle pressure. In a few days the discharge began to subside, and after eight days the dog was completely cured.

A bulldog suffered from phimosis of chronic character. In the course of several months it had been found repeatedly necessary to reduce the paraphimosis. When the dog came under my observation he presented, besides the above condition, an intense preputial catarrh with swelling of the foreskin. An operation was proposed by me for the relief of the phimosis, and was consented to by the owner. Before this could be done, however, it was necessary to remove the balanitis, and for this purpose I again employed protargol, at first in 3 per cent. solution. As this, however, proved ineffective I resorted at the end of eight days to a 6 per cent. solution. At the end of four weeks the catarrh was completely cured, and the operation could now be undertaken, the technique being as follows: The animal being anæsthetized the prepuce was split upon its dorsal surface upon a grooved director. The cut surfaces of the inner layer of the prepuce were sutured with silk to the corresponding surfaces of the external layer. Finally, from the inner layer there was formed a triangular flap which was stitched at the angle to the external layer. The operation was completely successful.

In a dog which, in consequence of purulent ophthalmia, developed a large corneal ulcer and presented also a very profuse catarrh of the conjunctiva, instillations of a few drops of a 1 per cent. protargol solution were made daily. At the end of fourteen days the eye was restored to a normal condition, except for the remaining cicatricial tissue, which was slight.

My colleague, Dr. Roth, has also employed protargol considerably in chronic cases of otitis and conjunctivitis, and has been so kind as to communicate to me his results, which are as follows:

Case 1. A St. Bernard, five years old, had suffered since 1899 from otitis externa, and had been treated for some time without success. The symptoms were a constant flapping of the ear, great sensitiveness to the touch, marked redness of the external meatus, and a profuse brownish secretion. The treatment consisted in the daily instillation of fifteen drops of a 3 per cent. protargol solution into the ear. As animals as a rule do not keep still a quantity was dropped in a small porcelain spoon and then poured in quickly. The ear was held closed, so that the fluid could not flow out, and a plug of cotton was then inserted. After eight days' use of this procedure the secretion ceased; the animal shook its head less, and the sensitiveness diminished. Instillations were then made at gradually longer intervals. After scarcely three weeks' treatment the disease, which had been present for two and one-half years, was completely cured.

Case 2. A St. Bernard, aged one and one-half years, had an otitis externa for six months, which was treated in the same manner as Case 1, and cured in about the same time.

Case 3. A shepherd dog, aged one and one-half years, presented the same symptoms as the above, and was cured in fourteen days.

Case 4. A German shepherd dog, aged two and one-half years, had an acute otitis externa of both ears, and was cured in sixteen days.

Case 5. A dog, aged four and one-half years, had otitis externa for some time. Besides the above symptoms there was a profuse and offensive discharge from both ears. After three days' treatment in the manner indicated the unpleasant odor disappeared and the secretion became less, and after eight days more a cure was obtained.

Case 6. A St. Bernard, aged four and one-half years, had an otitis externa for one-half year. The symptons consisted in a drooping of the left ear and frequent shaking of the head. The parts were extremely painful. The walls of the external auditory canal were covered with flat granulating surfaces which bled on the slightest touch. There was an abundant blackish discharge. The treatment was the same as in Case 1, the eroded places being covered with airol. After three weeks' daily treatment the wounds closed, the secretion was very slight, and the shaking of the head had ceased, and after thirteen days more a cure ensued.

Case 7. A St. Bernard, aged one and one-half years, with otitis externa, was cured in less than five weeks.

On the ground of these results Dr. Roth considers himself justified in stating that we possess in protargol an especially valuable remedy in otology, which, perhaps, surpasses all others in its rapid curative effect.

The two following observations may also prove of interest.

Case 8. A Scotch shepherd dog, aged nine months, suffered from an obstinate catarrhal conjunctivitis. The treatment consisted in two daily instillations of a $\frac{1}{2}$ per cent. protargol solution of three to five drops. After five days the suppuration was much diminished, and after four days more a cure was obtained.

Case 9. A fox terrier, aged one year, had a purulent preputial catarrh. This was cured in the course of thirteen days by injections of a $\frac{1}{2}$ per cent. protargol solution twice daily.

These observations, and especially those communicated by Dr. Roth in reference to protargol in the treatment of chronic otitis externa, are so encouraging as to incite to further experiments with the remedy.—*Wochenschrift für Thierheilkunde und Viehzucht*, No. 9, 1902.

